# Supplementary material for: The Use of an Antioxidant Enables Accurate Evaluation of the Interaction of Curcumin on Organic Anion-Transporting Polypeptides 4C1 by Preventing Auto-Oxidation
Source: Int J Mol Sci. 2024 Jan 12;25(2):991. doi: 10.3390/ijms25020991 (PMC10815578; doi:10.3390/ijms25020991)
Supplement: Supplementary file 1 [file ijms-25-00991-s001.zip › ijms-2747249-supplementary.pdf]

Supplementary Materials Data

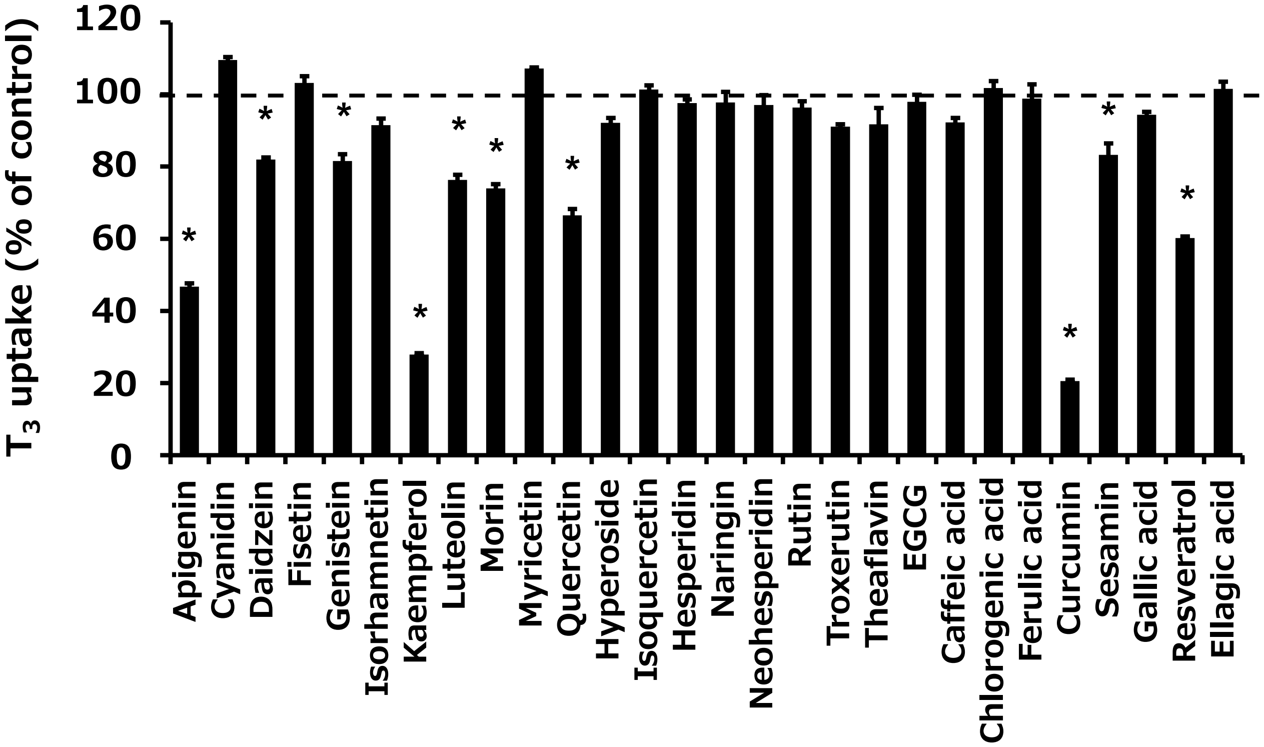

Scheme 1. Screening of the effect of food ingredients on OATP4C1-mediated transport under the presence of AA. Cells were incubated with 1 μM T_3 with or without food ingredients for 10 min at 37 °C under the presence of 10 μM AA. The concentrations of the compounds were 100 μM except for theaflavin (50 μM). The amount of OATP4C1-mediated uptake was calculated by subtracting the nonspecific uptake of T_3 by mock cells from the total cellular uptake by the OATP4C1-expressing cells. Data are shown as mean ± standard error (S.E.) (n = 3). *$p < 0.05$, significantly different from control by one-way analysis of variance followed by Tukey's test.

Scheme 1. Inhibition parameters of food ingredients for OATP4C1 and other drug transporters in the liver and kidney.

Inhibition (IC_{50} or K_i values (μM)) or elevation (EC_{50} values (μM)) parameters							
**Compounds**	**OATP4C1**	**OATP1B1**	**OATP1B3**	**OATP2B1**	**OAT1**	**OAT3**	P-gp
**Apigenin**	Yes (54%, 100 μM)	0.6 ± 0.2 [1*]<br>53.4 ± 2.1[1]<br>Yes [2]	16.7 ± 1.6 [1*]	13.9 ± 1.6 [3]<br>20.8 ± 8.0 [3]	0.212 ± 0.010 [4]	0.410 ± 0.157 [4]	-
**Cyanidin**	Yes (24%, 100 μM)	-	-	Yes [5]	-	-	-
**Daidzein**	Yes (40%, 100 μM)	-	-	22.3 ± 2.2 [6]<br>Yes [5]<br>20.7 ± 4.3 [6]	-	-	-
**Fisetin**	No	Yes [2]	-	-	Yes [7]	-	-
**Genistein**	Yes (42%, 100 μM)	14.9 ± 3.76 [2]	-	8.6 ± 1.8 [6]<br>Yes [5]<br>4.6 ± 2.5 [6]	-	-	Yes [8]
**Isorhamnetin**	Yes (18%, 100 μM)	3.12 [9]<br>46.12 ± 5.25 [10]	3.10 [9]	0.82 [9]	-	-	-
**Kaempferol**	25.1 ± 1.6*	16.0 ± 1.9 [1*]<br>32.4 ± 2.2 [1]<br>33.05 [11]<br>3.28 ± 1.08 [10]	24.4 ± 1.8 [1*]	15.1 ± 7.8 [3*]<br>20.7 ± 1.8 [3]<br>Yes [5]	-	-	Yes [12]
**Luteolin**	Yes (52%, 100 μM)	22.03 [11]<br>0.85 [13]<br>Yes [2]	-	Yes [5]<br>0.42 [13]	0.47 [7]<br>5.17 [14]	4.20 [14]	-
**Morin**	Yes (57%, 100 μM)	Yes [2]	-	-	< 0.3 [7]<br>0.5 [15]	-	Yes [16]
**Myricetin**	No	Yes [2]	-	-	Yes [7]	-	-
**Quercetin**	Yes (50%, 100 μM)	8.8 ± 0.8 [1*]<br>9.1 ± 1.0 [1]<br>15.9 ± 1.4 [17]<br>1.55 [9]<br>3.28 ± 1.08 [10]<br>1.82 [18]	7.8 ± 1.7 [1*]<br>3.22 [9]	8.7 ± 0.1 [3*]<br>14.1 ± 1.3 [3]<br>Yes [5]<br>0.34 [9]<br>7.5 ± 1.2 [19]	Yes [7]	-	Yes [20]
**Hyperoside**	No	-	-	29.4 ± 1.5[19]	-	-	-
**Hesperidin**	No	-	-	Yes [5]<br>72.3 ± 1.4 [19]<br>>300 [21]	-	-	-
**Naringin**	No	Yes<br>(<50%, 250 μM) [1]	197.5 ± 1.1 [1]	8.3 ± 1.2 [22]<br>37.0 [23]<br>36.4 ± 8 [21]<br>6.9 ± 1.4 [22]	-	-	Yes [24]
**Rutin**	No	Yes<br>(<50%, 150 μM) [1]<br>Elevation<br>(81.1%, 50 μM) [2]	111.5 ± 1.1 [1]	60.7 ± 1.2 [19]	-	-	Yes [20]
**Theaflavin**	No	-	-	8.2 ± 1.7 [5*]	-	-	Yes [20]
**EGCG**	No	14.1 ± 1.4 [2]<br>7.8 ± 0.9 [25]	Elevation<br>(EC_{50}=10.5 ± 1.6) [25]<br>8.4±0.6 [25]	101±9 [25]<br>Yes [26]	334 ± 58 [27*]	162 ± 29 [27*]	Yes [20]
**Caffeic acid**	No	-	-	-	5.22 [28]<br>16.6 ± 3.7 [29]	30.8 [28]<br>5.4 ± 1.3 [29]	Yes [30]
**Ferulic acid**	No	-	-	-	9.01 [28]	7.35 ± 3.73 [31]	Yes [32]
**Curcumin**	23.5 ± 0.5*	3.81 [33]	33.7 [33]	Yes [5]	-	-	Yes [34]
**Gallic acid**	No	-	1.60 ± 0.60 [35]	-	1.24 ± 0.36 [31]<br>3.73 [36]	9.02 ± 3.24 [31]<br>29.41 [36]	Yes [37]
**Resveratrol**	Yes (46%, 100 μM)	9.1 [38]	11.6 [38]	8.3 [38]	Yes [39]	Yes [39]	6.64 [40]<br>Yes [39]
**Ellagic acid**	Elevation<br>(13%, 100 μM)	-	-	-	0.207 [41]	-	Yes [37]

K_i values of OATP4C1 are expressed as mean ± S.E. Data for IC_{50}, K_i, or EC_{50} values of the food ingredients are obtained from previous reports. Asterisk (*) indicates a K_i value instead of an IC_{50}

value. EC_{50} value is expressed as mean ± S.E. if the compound elevates the transporter mediated uptake. Yes, the food ingredients inhibited the transporter-mediated substrate uptake (inhibition degree, concentration of the flavonoid (if available)); No, no inhibition or elevation was observed; -, no data available; Elevation, elevation was observed.
